# Non-Hodgkin’s lymphoma relapse as Burkitt lymphoma leading to ileocecal intussusception in an adult patient—a case report

**DOI:** 10.1093/jscr/rjae087

**Published:** 2024-02-22

**Authors:** Mohsin Yahya Murshid, Anfal Nawawi, Ahmad Jan, Rafat I Abu Shakra

**Affiliations:** Department of General Surgery, International Medical Center, P.O. Box 2172, Jeddah 21451, Saudi Arabia; Department of General Surgery, International Medical Center, P.O. Box 2172, Jeddah 21451, Saudi Arabia; Department of General Surgery, International Medical Center, P.O. Box 2172, Jeddah 21451, Saudi Arabia; Department of Pathology & Lab Med, International Medical Center, P.O. Box 2172, Jeddah 21451, Saudi Arabia

**Keywords:** Burkitt lymphoma, non-Hodgkin lymphoma, intussusception, gastrointestinal lymphoma, adult intussusception, ileocecal valve

## Abstract

Burkitt Lymphoma, an aggressive form of non-Hodgkin’s lymphoma, is a rare cause of ileocolic intussusception in adult patients. We present the case of a 17-year-old male patient, with a history of non-Hodgkin’s lymphoma in childhood, presenting with acute abdominal pain, vomiting, and diarrhea. CT and colonoscopy findings revealed ileocolic intussusception with a large ileocecal mass, leading to a diagnosis of Burkitt Lymphoma after histopathological and immunohistochemical examination. This case highlights the rarity of Burkitt Lymphoma causing intussusception in adults, a condition more commonly seen in children. The case also underscores the importance of considering Burkitt Lymphoma in patients with a history of non-Hodgkin’s lymphoma presenting with acute abdominal symptoms. He was successfully treated with surgery without any complications. On follow-ups, he is doing well.

## Introduction

Burkitt’s lymphoma, an aggressive and rapidly growing type of malignant non-Hodgkin’s lymphoma (NHL) that primarily affects B cells, is one of the rare underlying causes of intussusception [1]. Burkitt lymphoma has been diagnosed in a wide range of extranodal sites, including the ileocecal region where it may cause intestinal obstruction by either indirect pressure or direct luminal involvement. Intussusception is the presenting feature in 18% of the patients with primary abdominal Burkitt lymphoma [2]. Intussusception is common in children and is the second most common cause of abdominal emergency in children with 90% of cases are idiopathic in nature [3]. It is relatively rare in adults and unlike children, exhibit distinct clinical manifestations, thus resulting in difficulty in diagnosis. These cases in adults are mostly attributed to a distinct disease, such as carcinomas, polyps, and benign neoplasms, acting as a lead point [4]. Our report details a case of relapse of non-Hodgkin lymphoma manifesting as Burkitt Lymphoma presenting with ileocolic intussusception in an adult patient.

## Case report

**Figure 1:**
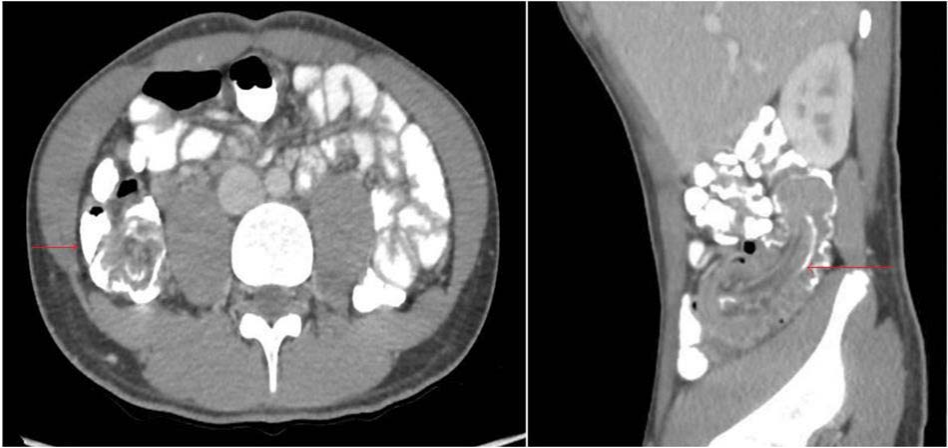
CT Abdomen (axial and sagittal plane) showing ileocolic intussusception extending for 10 cm with a soft tissue lesion at the tip of the intussusceptum concerning underlying lead point with reactive mesenteric lymph nodes.

A 17-year-old male presented to the Emergency Department with a 4-day history of abdominal pain localized to the peri-umbilical area. The patient started experiencing the symptoms 3 days before the presentation. The pain was colicky, accompanied by multiple episodes of vomiting and diarrhea. The patient had a previous history of NHL at the age of 4, which manifested as neck mass. He had undergone six cycles of chemotherapy and had been in remission without any signs of relapse until his current presentation. The patient had no significant family history. The patient was conscious and stable at the time of presentation. On physical examination, the patient had tenderness in the right lower quadrant. The patient’s laboratory results were unremarkable: hematocrit was normal, white blood cell count was 6.77 × 103/μL, C-reactive protein level was 0.41 mg/dL, creatinine level was 0.59 mg/dL, urea level was 21 mg/dL, and aspartate aminotransferase level was 15 U/L. CT Abdomen revealed non-obstructive ileocolic intussusception extending for 10 cm with a soft tissue lesion at the tip of the intussusceptum concerning underlying lead point with reactive mesenteric lymph nodes (Fig. 1).

**Figure 2:**
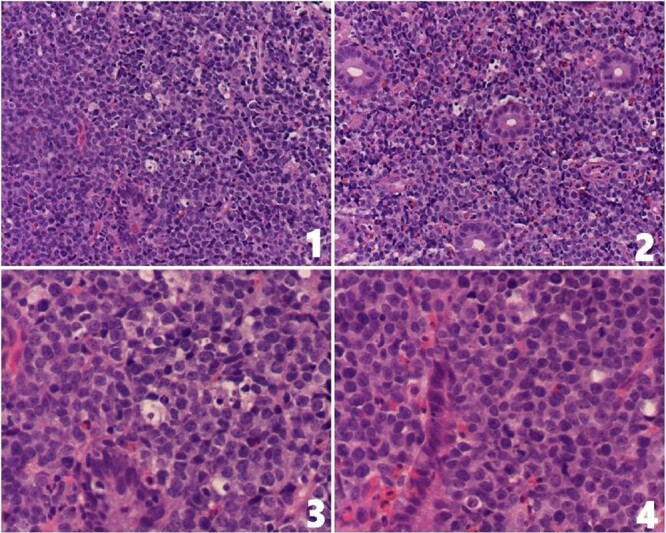
(1 and 2) Ileocecal mucosa showing infiltration by monotonous medium-sized lymphocytes displaying fine chromatin pattern and small nucleoli; tingible body macrophages are seen (H&E stain, 20×); (3 and 4) high-power magnification showing infiltration by monotonous medium-sized lymphocytes displaying fine chromatin pattern and small nucleoli. Apoptotic bodies and tingible body macrophages are present (H&E stain, 40×).

**Figure 3:**
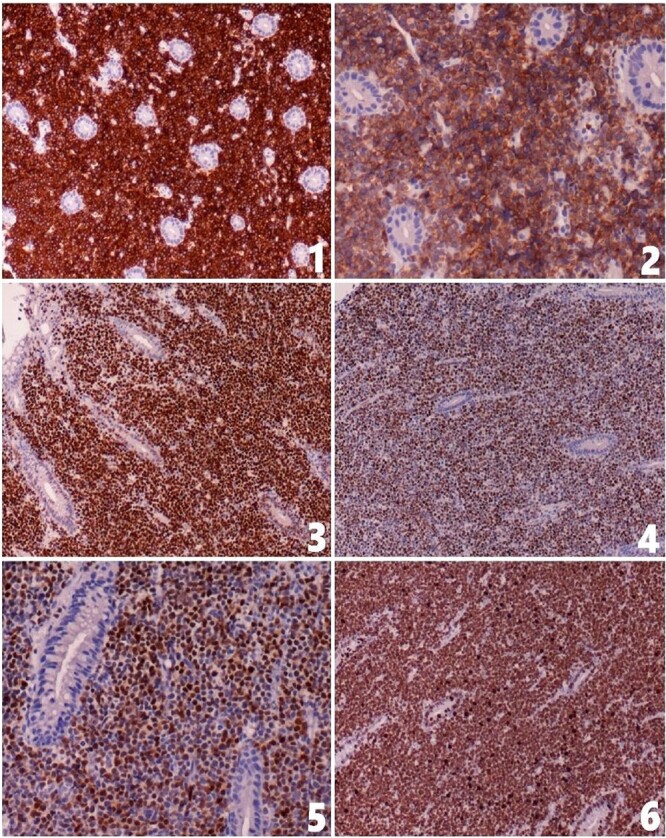
The tumor cells show positive staining for (1) CD79a, (2) CD10, (3) MYC, (4) BCL6 and (5) MUM1; (6) the Ki67 index is about 100%.

A colonoscopy was performed for its potential in therapeutic intervention, diagnosis, and tissue sampling. The colonoscopy revealed a large ileocecal mass measuring around 4 × 3 cm, characterized as friable, fungating, and solid, causing complete obstruction at the ileocecal valve. Biopsy samples were taken, and multiple small polyps were observed in the ascending, transverse, descending, and sigmoid colon. After discussing the endoscopic findings and potential management options with the family, an urgent surgical resection plan was agreed upon. The patient underwent a laparoscopic right hemicolectomy with ileo-colic anastomosis along with regional lymphadenectomy. The histopathological examination of the resected specimen indicated the features of a B-cell immune-phenotype, high-grade NHL suggesting a diagnosis of Burkitt’s lymphoma (Fig. 2). The immunohistochemistry testing yielded positive results for CD79a, CD10, MYC, BCL6, and MUM1. The Ki67 index was 100% as shown in Fig. 3. The patient’s postoperative course was uneventful. Patient’s recovery was satisfactory. The patient was subsequently referred to an oncologist for further management.

## Discussion

Primary gastrointestinal lymphomas account for 1–4% of all gastrointestinal cancers, with Burkitt’s lymphoma comprising 0.3–1.3% of all NHLs [5]. It is a common lead point in intussusception cases, reported to be as high as 17% generally and over 50% in children aged 4–6 years. However, its occurrence in older age groups is rare [6–8]. Burkitt’s lymphoma is a rapidly growing, highly aggressive variant of NHL. Its incidence is estimated at 0.7 per million in the 10–20 age group. There are 4 variants: Endemic, largely found in Africa; Sporadic, found outside Africa and primarily affecting abdominal organs; immunodeficiency associated; and type of posttransplant lymphoproliferative disorders [9–11].

Intussusception due to Burkitt lymphoma in adult patients is extremely rare. The symptoms often mislead, complicating the diagnosis. The rapid growth rate of this neoplasm can lead to acute abdomen presentations that mimic other, more common diseases. The adult intussusception accounts for only 5% of total cases and causes 1–5% of all cases of intestinal obstructions. Intussusceptions can be classified as benign or malignant, depending on their etiology [12].

The literature significantly lacks reports on intussusception caused by Burkitt’s lymphoma in adults. Kulendran *et al.* described a case akin to ours, involving a 15-year-old boy with ileocolic intussusception due to Burkitt’s lymphoma. Notably, their patient experienced severe abdominal pain, vomiting, and diarrhea for 3 weeks, contrasting with our case’s timeline which presented acutely within 72 hours of onset [13].

In adolescents and adults presenting with severe abdominal pain, accompanied by nausea and vomiting, there should be a high index of suspicion for intussusception. If imaging reveals ileocecal intussusception, radiological reduction might not be as effective, making colonoscopy a necessary step. Besides potentially providing therapeutic benefits, colonoscopy also serves as a definitive tool for evaluating findings from imaging. It can also help identify the exact cause of the intussusception by allowing for the biopsy of any lesions, such as polyps or tumors, for histological examination.

The management of abdominal Burkitt lymphoma requires a multidisciplinary approach that includes both surgical and oncological interventions. Surgery plays a crucial role in treating Burkitt’s lymphoma, both for confirming the diagnosis and alleviating symptoms such as intestinal obstruction, abdominal mass, or intussusception. Achieving complete resection of the tumor is linked to better survival outcomes. Studies have shown a higher survival rate, ranging from 58 to 89%, in patients who undergo extensive surgical resection, compared with a 40–45% survival rate in those who have only partial or incomplete resection, over a period of 2–5 years [10, 14].

## Conclusion

This case report emphasizes the rare development of Burkitt Lymphoma due to NHL relapse, presenting as intussusception. It suggests considering Burkitt Lymphoma in patients with prior NHL and acute abdominal symptoms. Surgical resection is the treatment of choice.

## Conflict of interest statement

None declared.

## Funding

None declared.

## Consent

Informed written consent was obtained from the patient’s father for publication of this report and any accompanying images.
